# Utility and Safety of ERCP in the Elderly: A Comparative Study in Iran

**DOI:** 10.1155/2012/439320

**Published:** 2012-07-09

**Authors:** Amir Houshang Mohammad Alizadeh, Esmaeil Shamsi Afzali, Anahita Shahnazi, Azare Sanati, Dariush Mirsattari, Mohammad Reza Zali

**Affiliations:** Research Center for Gastroenterology and Liver Diseases, Taleghani Hospital, Shaheed Beheshti University of Medical Sciences and Health Services, Parvaneh Avenue, Tabnak Street, Evin, P.O. Box 19835-178, Tehran 1985717413, Iran

## Abstract

*Background*. The present study sought and compared the utility and safety of endoscopic retrograde cholangiopancreatography (ERCP) in the elderly and younger people in a great sample of Iranian population. *Methods*. Our study involved 780 patients undergoing diagnostic and therapeutic ERCP at the Taleghani hospital in Tehran between 2010 and 2011; among them, 558 patients were less than 70 years old and others were 70 years old or older. The patients were prospectively identified and data including clinical and biochemical features, ERCP procedures, ERCP diagnosis, and ERCP complications were gathered on them prospectively. *Results*. Clinical manifestations were comparable in young and older groups except for hepatosplenomegaly and constipation that were more prevalent in the elderly. Laboratory findings were similar in both groups other than mean levels of alkaline phosphatase, hemoglobin and albumin levels, which were higher in the elderly group. Selective biliary cannulation was technically more successful in the younger than in others (89.0% versus 81.8%). Common bile duct stone was the most frequent diagnosis in both young and older groups (36.6% and 45.9%, resp.), whereas ERCP was reported to be normal in 13.4% of the younger and 5.0% of the elderly patients. Post-ERCP complications were observed in 4.8% of patients aged less than 70 years in comparison with 2.3% of patients aged over 70 years. The most frequent complication was pancreatitis that was significantly more developed in young than older patients (3.6% versus 1.5%; OR: 8.216, *P* = 0.015). *Conclusion*. Diagnostic ERCP is safe and well tolerated in the elderly and even associated with significantly less risk than the younger.

## 1. Introduction

Disorders affecting the gall bladder and biliary ducts occur commonly in the elderly and lead to choledocholithiasis in most of this age population. While the prevalence of biliary disorders in the men and women in the age group lower than 30 years is low, its sharply increased prevalence in men older than 60 years and women older than 50 years especially in developing countries is estimated more than 10-fold. However, this does not reach the high prevalence seen in Western countries [1]. Untimely diagnosis and management of biliary tract pathologies and delay in giving appropriate treatment for these defects can potentially increase the risks of their related complications. This subject is more vital and important in older patients with atypical clinical manifestations [2]. 

Although endoscopic retrograde cholangiopancreatography (ERCP) has been confirmed as a widely used technique for the diagnosis and management of biliary obstruction, its safety and efficacy in the elderly have been already questioned. Among reports involving ERCP in elderly patients, some found the procedure to be of high diagnostic value and achieved a 92% clearance of common bile duct stones following this therapeutic procedure [3]. Some researchers believe that the post-ERCP mortality and complications are mainly due to the type of sedation, the severity of illness, or underlying malignancies [4]. In addition, some others showed that the complications following ERCP were similar between the young patients and the elderly [5, 6]. Therefore, despite the high probable comorbidity of ERCP in older patients, its outcome can be acceptable and biliary cannulation can be successfully achieved in advanced ages.

However, a clarification of the efficacy and accuracy of ERCP in the elderly is needed. In addition, no previous population-based report is available on the success rate and outcome of ERCP in Iranian elderly patients. The goal of this study was to evaluate the safety and efficacy of ERCP in the diagnosis of biliary stones in patients aged 70 years or older compared with the younger patients in a great sample of Iranian population. 

## 2. Materials and Methods

Baseline and post-ERCP information of all patients with hepatobiliary disease of the biliary tract referred to the liver services of Taleghani hospital in Tehran was evaluated and organized in a computerized database. All patients signed research study informed consent documents before ERCP procedure, and the study protocol was approved by the ethics committee of the internal review board of Shaheed Beheshti University of Medical Sciences and Health Services. The current study involved 780 patients undergoing diagnostic and therapeutic ERCP at the Taleghani hospital in Tehran between 2010 and 2011; among them, 558 patients were less than 70 years old and others were 70 years old or older. Due to lower life expectancy of Iranian population compared to developed and industrialized countries and low frequency of patients above 80 years in our study, age 70 was considered as comparison level. Data including demographic characteristics and medical history, clinical and biochemical features, and ERCP findings and its related complications were gathered on them from recorded files or by interviewing on the day of admission to hospital. Patients with a history of biliary sphincterotomy or precut sphincterotomy, preprocedure active pancreatitis, pregnancy, mental disability, and refusal to participate were excluded. The first liver function tests results during the acute admission were used as the screening tests for ERCP. Other laboratory parameters were also measured on the day of admission. All patients underwent ERCP for suspected and diagnosed pancreatobiliary disease and on the basis of generally accepted diagnostic indications for ERCP [7]. Similar procedure was done in all patients. Procedure was performed under conscious sedation with midazolam and meperidine and by a single staff gastroenterologist. Cannulation was performed on the basis of techniques as previously described [8]. Successful cannulation was defined as free and deep instrumentation of the biliary tree, and a cannulation attempt was defined as sustained contact with the cannulating device and the papilla for at least five seconds [9]. Difficult biliary cannulation was also related to the failure of biliary access despite ten minutes of attempted biliary cannulation, or more than five attempted unintentional pancreatic cannulations [10]. Post-ERCP complications including at least one of these: post-ERCP pancreatitis, gastrointestinal perforation and bleeding. 

Results were expressed as the mean ± standard deviation (SD) for quantitative variables and percentages for categorical variables. Categorical variables between the variables were compared using *χ* ^2^ square test or Fisher's exact test. Continuous variables were compared by independent samples *t*-test for variables with normal distributions and Mann-Whitney test for variables with nonnormal distributions.  The role of advanced age for predicting common bile duct stone and also biliary cannulation failure was assessed by multivariable linear regression analysis adjusting for confounders. *P* values of 0.05 or less were considered statistically significant. All the statistical analyses were performed using SPSS version 16.0 (SPSS Inc., Chicago, IL, USA) and SAS version 9.1 for Windows (SAS Institute Inc., Cary, NC, USA). 

## 3. Results

At baseline (Table 1), the younger and older groups were similar in terms of sex ratio, diabetes mellitus, history of previous ERCP, and cholecystectomy as well as history of biliary stone and cirrhosis. There were also no significant differences in the overall incidence rates for current cigarette smoking, alcohol use, and opium addiction between the two age groups. Clinical manifestations were comparable in young and older groups except for hepatosplenomegaly and constipation that were more prevalent in the elderly (Table 2). Furthermore, regarding laboratory parameters, findings were similar in both groups other than mean levels of alkaline phosphatase, hemoglobin and albumin levels, which were higher in the elderly group (Table 3). 

**Figure 1:**
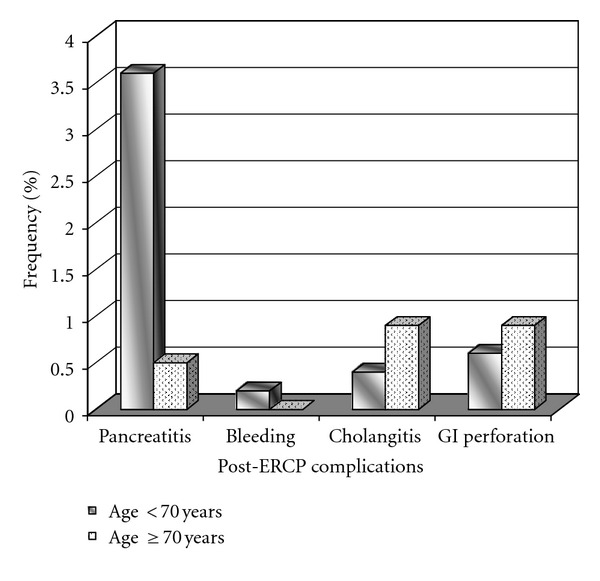


Successful biliary cannulation was technically achieved in 81.8% of the patients ≥ 70 years old and in 89.0% of other patients (*P* < 0.05), that is in 14.9% and 11.0% of them there was difficulty while performing, respectively (Figure 1). Cannulation of common bile duct failed more in patients aged 70 years or older compared to others (18.2% versus 10.7, *P* = 0.005). The difference related to failed cannulation between the younger and older patients was also confirmed by  multivariable logistic regression analysis with the presence of potential confounders such as hypertension, coronary artery disease, serum total bilirubin, and history of bile duct stone or cholecystectomy (OR = 1.817, 95% CI = 1.031–3.201, *P* = 0.039). Common bile duct stone was the most frequent diagnosis in the young and older groups (36.6% versus 45.9%, *P* = 0.016), whereas ERCP was reported normal in 13.4% of the younger and 5.0% of the elderly patients (*P* < 0.001). Multivariable analysis also showed that the participants ≥ 70 years old had about two times more often common bile duct stone in comparison with the younger patients (OR = 1.685, 95% CI = 1.185–2.396, *P* = 0.004).

Malignancy was more slightly frequent as a cause of biliary obstruction in patients above 7 years old (12.0% versus 17.1%; *P* = 0.059). Post-ERCP complications were observed in 4.8% of patients aged less than 70 years in comparison with 2.3% of patients aged over 70 years. The most frequent complication was pancreatitis that was significantly more developed in young than older patients (3.6% versus 1.5%; OR: 8.216, *P* = 0.015). However, other complications were found similar in the two groups (Figure 1). 

## 4. Discussion 

The current study had some important findings that are comparable with other recent studies. First, except for hepatosplenomegaly and constipation that were observed more in the elderly, other manifestations were not different between the two age groups. Furthermore, regarding laboratory indices, the mean level of serum alkaline phosphatase was significantly higher in the elderly. It has been clear that the classical presentations for CBD  obstructions due to biliary stone including abdominal pain, with or without jaundice, and Charcot's triad occurred in only half the patients, whereas nonspecific presentations such as constipation or splenomegaly can occur in almost 43% of them [3], supporting the view that the presentation of CBD stones is often atypical in elderly patients compared with the younger. 

In our study, although successful biliary cannulation was achieved in 89.0% of other patients, cannulation failure rate in these participants was notably high (18.2%). Fritz et al. achieved a successful cannulation in 88% of older patients [11]. Also, in Köklü et al.'s study, selective biliary cannulation was technically successful in 99% of both young and older groups [12]. In other reports, success rate of ERCP has been shown to be acceptable in old patients especially in the West [13–16]. The discrepancy between the reported successful cannulation can be the result of differences in operator experience for performing this procedure. 

Postprocedure complication was rarely observed in patients undergoing ERCP and is estimated to be about 5–10% [17–19]. In the present series, most of the complications were minor and none of them resulted in post-ERCP death. Pancreatitis was the most frequent complication that was observed more than two times in the younger than in older group. The most common minor post-ERCP complications include minor events such as tachycardia, desaturation, transient hypotension, self-limiting bleeding, extravasations, and mild pancreatitis. Furthermore, major events are reported as postsphincterotomy bleeding, duodenal perforation, cholangitis, and severe pancreatitis [4]. In the general population, the most common ERCP-related complication is pancreatitis. The reported incidence of this complication varies from less than 1% to 40%, but a rate of 4%–8% is reported in most prospective studies involving nonselected patients [20]. We showed that except for pancreatitis that was more prevalent in the younger, other complications were comparable between the two age groups. This result has been also reported by other previous studies [11, 21, 22]. In the last decade, great efforts have been exerted toward the prevention of this complication. Points of emphasis have included technical measures, pharmacological prophylaxis, and patient selection [20, 23]. It seems that the role of patient factors such as age and prior history of post-ERCP pancreatitis as well as technical factors such as number of minor papilla sphincterotomy and operator experience as the determining high-risk predictors for post-ERCP pancreatitis is highlighted.

## 5. Conclusion

In conclusion, diagnostic ERCP is safe and well tolerated in the elderly. Post-ERCP events were frequently minor and comparable in the young and older patients except for pancreatitis that may have occurred more in the younger. Successful biliary cannulation is achieved in most of the elderly and can be restricted to those with underlying disorders and risk factors. Finally, advanced age should not be an exclusion index for selecting patients for ERCP.

## Figures and Tables

**Table 1:** Baseline characteristics and medical history in the group aged less than 70 years in comparison with the elderly.

Characteristics	Group < 70 years old (*n* = 558)	Group ≥ 70 years old (*n* = 222)	*P* value
Male gender	270 (48.4)	123 (55.7)	0.067
Age (years)	49.6 ± 14.0	76.3 ± 5.0	<0.001
Medical history:			
Diabetes mellitus	69 (12.4)	20 (9.0)	0.183
Hypertension	78 (14.0)	63 (28.4)	<0.001
Coronary artery disease	32 (5.7)	31 (14.0)	<0.001
Cholecystectomy	207 (37.1)	76 (34.2)	0.453
Previous ERCP	51 (9.1)	19 (8.6)	0.798
Biliary stone	56 (10.0)	24 (10.8)	0.748
Cirrhosis	8 (1.4)	3 (1.4)	0.999
Pancreatitis	24 (4.3)	3 (1.4)	0.042
Cigarette smoking	66 (11.8)	31 (14.0)	0.415
Alcohol use	17 (3.0)	5 (2.3)	0.545
Opium addiction	29 (5.2)	13 (5.9)	0.713

Data are presented as mean ± SD or *n* (%).

**Table 2:** Clinical manifestations in the group aged less than 70 years in comparison with the elderly.

Manifestations	Group < 70 years old (*n* = 558)	Group ≥ 70 years old (*n* = 222)	*P* value
Fever	25 (4.5)	15 (6.8)	0.193
Weight loss	165 (29.6)	78 (35.1)	0.130
Anorexia	136 (24.4)	57 (25.7)	0.704
Fatigue	40 (7.2)	25 (11.3)	0.062
Icterus	267 (47.8)	102 (45.9)	0.631
hepatosplenomegaly	9 (1.6)	10 (4.5)	0.018
Dark urine	152 (27.2)	58 (26.1)	0.752
Diarrhea	5 (0.9)	5 (2.3)	0.158
Constipation	39 (7.0)	27 (12.2)	0.019

Data are presented as *n* (%).

**Table 3:** Pre-ERCP laboratory parameters in the group aged less than 70 years in comparison with the elderly.

Laboratory parameters	Group < 70 years old (*n* = 558)	Group ≥ 70 years old (*n* = 222)	*P* value
Hemoglobin	12.0 ± 1.8	11.6 ± 1.7	0.030
Albumin	4.0 ± 0.6	3.7 ± 0.6	<0.001
ESR	31.7 ± 18.4	32.1 ± 18.7	0.776
AST	85.5 ± 95.1	86.3 ± 80.6	0.907
ALT	103.5 ± 114.5	125.9 ± 330.7	0.338
ALP	784.1 ± 750.1	919.0 ± 888.9	0.038
Lactate dehydrogenase	472.3 ± 361.0	444.7 ± 225.6	0.364
Total bilirubin	6.4 ± 8.8	6.5 ± 9.0	0.815
Direct bilirubin	3.5 ± 5.1	3.7 ± 5.3	0.640
Serum baseline amylase	160.1 ± 345.5	190.1 ± 365.2	0.402

Data are presented as mean ± SD.

## References

[1] Massarrat S (2001). Prevalence of gallstone disease in Iran. *Journal of Gastroenterology and Hepatology*.

[2] Affronti J (1999). Biliary disease in the elderly patient. *Clinics in Geriatric Medicine*.

[3] Ashton CE, Robin Mcnabb W, Wilkinson ML, Lewis RR (1998). Endoscopic retrograde cholangiopancreatography in elderly patients. *Age and Ageing*.

[4] Chong VH, Yim HB, Lim CC (2005). Endoscopic retrograde cholangiopancreatography in the elderly: outcomes, safety and complications. *Singapore Medical Journal*.

[5] Freeman ML, Nelson DB, Sherman S (1996). Complications of endoscopic biliary sphincterotomy. *The New England Journal of Medicine*.

[6] Deans GT, Sedman P, Martin DF (1997). Are complications of endoscopic sphincterotomy age related?. *Gut*.

[7] Silviera ML, Seamon MJ, Porshinsky B (2009). Complications related to endoscopic retrograde cholangiopancreatography: a comprehensive clinical review. *Journal of Gastrointestinal and Liver Diseases*.

[8] Lee TH, Park DH, Park JY (2009). Can wire-guided cannulation prevent post-ERCP pancreatitis? A prospective randomized trial. *Gastrointestinal Endoscopy*.

[9] Bailey AA, Bourke MJ, Williams SJ (2008). A prospective randomized trial of cannulation technique in ERCP: effects on technical success and post-ERCP pancreatitis. *Endoscopy*.

[10] Kaffes AJ, Sriram PVJ, Rao GV, Santosh D, Reddy DN (2005). Early institution of pre-cutting for difficult biliary cannulation: a prospective study comparing conventional vs. a modified technique. *Gastrointestinal Endoscopy*.

[11] Fritz E, Kirchgatterer A, Hubner D (2006). ERCP is safe and effective in patients 80 years of age and older compared with younger patients. *Gastrointestinal Endoscopy*.

[12] Köklü S, Parlak E, Yüsel O, Sahin B (2005). Endoscopic retrograde cholangiopancreatography in the elderly: a prospective and comparative study. *Age and Ageing*.

[13] MacMahon M, Walsh TN, Brennan P, Osborne H, Courtney MG (1993). Endoscopic retrograde cholangiopancreatography in the elderly: a single unit audit. *Gerontology*.

[14] Clarke GA, Jacobson BC, Hammett RJ, Carr-Locke DL (2001). The indications, utilization and safety of gastrointestinal endoscopy in an extremely elderly patient cohort. *Endoscopy*.

[15] Bergman JJGHM, Rauws EAJ, Tijssen JGP, Tytgat GNJ, Huibregtse K (1995). Biliary endoprostheses in elderly patients with endoscopically irretrievable common bile duct stones: report on 117 patients. *Gastrointestinal Endoscopy*.

[16] Mitchell RMS, O’Connor F, Dickey W (2003). Endoscopic retrograde cholangiopancreatography is safe and effective in patients 90 years of age and older. *Journal of Clinical Gastroenterology*.

[17] Loperfido S, Angelini G, Benedetti G (1998). Major early complications from diagnostic and therapeutic ERCP: a prospective multicenter study. *Gastrointestinal Endoscopy*.

[18] Masci E, Toti G, Mariani A (2001). Complications of diagnostic and therapeutic ERCP: a prospective multicenter study. *American Journal of Gastroenterology*.

[19] Rabenstein T, Schneider HT, Bulling D (2000). Analysis of the risk factors associated with endoscopic sphincterotomy techniques: preliminary results of a prospective study, with emphasis on the reduced risk of acute pancreatitis with low-dose anticoagulation treatment. *Endoscopy*.

[20] Adbel Aziz AM, Lehman GA (2007). Pancreatits after endoscopic retrograde cholangio-pancreatography. *World Journal of Gastroenterology*.

[21] Ávila-Funes JA, Montaño-Loza A, Zepeda-Gómez S (2005). Endoscopic retrograde cholangiopancreatography in the elderly. *Revista de Investigacion Clinica*.

[22] Talar-Wojnarowska R, Szulc G, Woźniak B, Pazurek M, Małecka-Panas E (2009). Assessment of frequency and safety of endoscopic retrograde cholangiopancreatography in patients over 80 years of age. *Polskie Archiwum Medycyny Wewnetrznej*.

[23] Cheng CL, Sherman S, Watkins JL (2006). Risk factors for post-ERCP pancreatitis: a prospective multicenter study. *American Journal of Gastroenterology*.

